# Enteroscopy-guided retrieval of a migrated gastric bypass stent from the mid-to-distal jejunum

**DOI:** 10.1055/a-2712-3652

**Published:** 2025-11-04

**Authors:** Si Chen, Wenyu Li, Tianying Duan

**Affiliations:** 1 70566 Department of Gastroenterology, The Second Xiangya Hospital of Central South University, Changsha, China; 2 12570 Research Center of Digestive Disease, Central South University, Changsha, China; 3 Clinical Research Center for Digestive Diseases in Hunan Province, Changsha, China

A 30-year-old man who underwent endoscopic duodenal-jejunal bypass stent placement two months ago for weight loss was admitted to the hospital complaining of abdominal pain and episodic vomiting. Esophagogastroduodenoscopy revealed fresh blood stains in the duodenal bulb, while the bypass stent was not observed. A computed tomography (CT) scan showed a dense shadow in the middle segment of the jejunum ( Fig. 1 ).

**Fig. 1:**
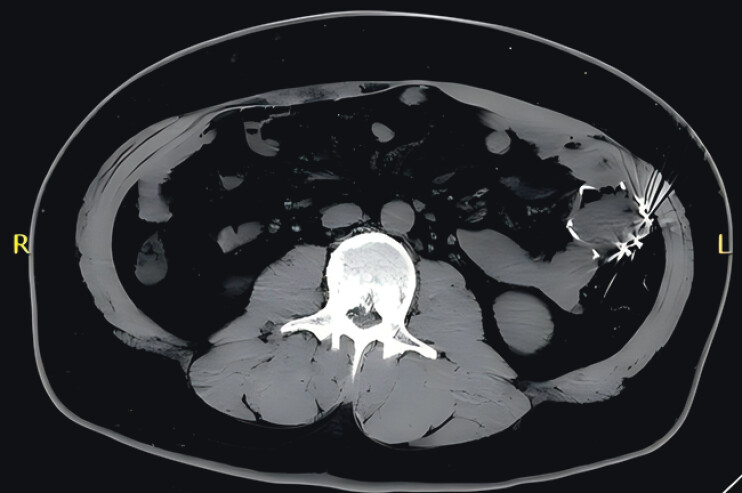
Computed tomography scan showed the stent had migrated to the jejunum.

In order to retrieve the bypass stent, a double-balloon enteroscopy was performed with a distal attachment cuff mounted on the scope tip. When the scope was advanced to the mid-to-distal jejunum, the gastric bypass stent was identified. The stent was well-positioned without overturning, and the retrieval loop was clearly accessible ( Fig. 2 ). A 2.6-meter retrieval hook was used to grasp the loop. By retracting the hook handle, the stent was gathered into a bundle. After confirming complete encapsulation, the stent bundle was fully retracted into the attached cuff and withdrawn slowly under the endoscopic guidance ( Video 1 ). Repeat enteroscopy revealed a superficial ulcer at the prior stent site, with no active bleeding noted after irrigation.

**Fig. 2:**
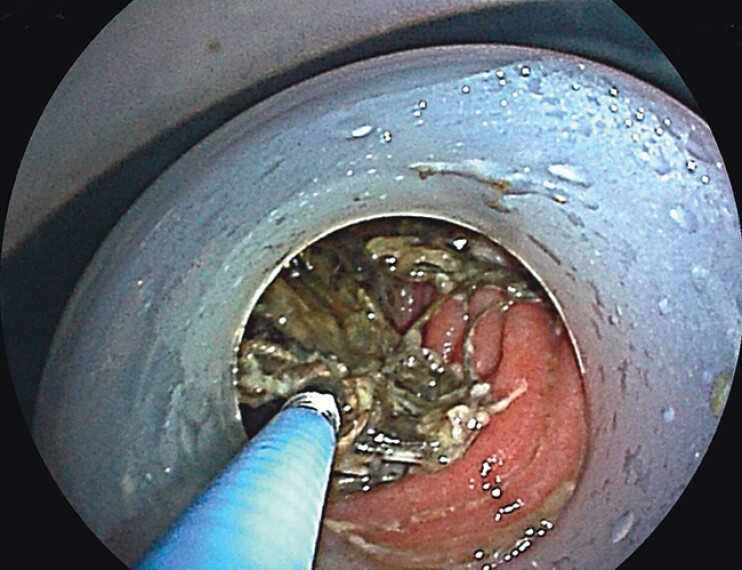
Enteroscopy revealed the gastric bypass stent had migrated to the mid-to-distal jejunum.

A migrated gastric bypass stent was successfully removed by a retrieval hook under enteroscopy guidance.Video 1

A gastric bypass stent was first reported for treatment of obese patients with nonalcoholic fatty liver disease in 2023 1 . It is used increasingly in China because it is a simple, minimally invasive operation with advantages over bypass gastrectomy 2 . In this report, we first described a patient with migration of a gastric bypass stent that was successfully removed by a retrieval hook under enteroscopy guidance. It reminded us that postoperative surveillance following a bypass procedure is crucial for early detection of stent migration and to prevent life-threatening complications of hemorrhage and perforation. Regarding the complication of stent migration, the enteroscopy-assisted retrieval approach could be considered as a first-line therapeutic strategy for its minimally invasive profile and avoidance of further surgical intervention.

Endoscopy_UCTN_Code_TTT_1AP_2AD

## Notes

Endoscopy E-Videos https://eref.thieme.de/e-videos
*E-Videos* is an open access online section of the journal *Endoscopy* , reporting on interesting cases and new techniques in gastroenterological endoscopy. All papers include a high-quality video and are published with a Creative Commons CC-BY license. Endoscopy E-Videos qualify for HINARI discounts and waivers and eligibility is automatically checked during the submission process. We grant 100% waivers to articles whose corresponding authors are based in Group A countries and 50% waivers to those who are based in Group B countries as classified by Research4Life (see: https://www.research4life.org/access/eligibility/ ). This section has its own submission website at https://mc.manuscriptcentral.com/e-videos .
